# The role of artificial intelligence in healthcare: a structured literature review

**DOI:** 10.1186/s12911-021-01488-9

**Published:** 2021-04-10

**Authors:** Silvana Secinaro, Davide Calandra, Aurelio Secinaro, Vivek Muthurangu, Paolo Biancone

**Affiliations:** 1 Department of Management, University of Turin, Turin, Italy; 2 Ospedale Pediatrico Bambino Gesù, Rome, Italy; 3 Institute of Child Health, University College London, London, UK

**Keywords:** Artificial intelligence, Healthcare, Patient data, Clinical decision-making, Management

## Abstract

**Background/Introduction:**

Artificial intelligence (AI) in the healthcare sector is receiving attention from researchers and health professionals. Few previous studies have investigated this topic from a multi-disciplinary perspective, including accounting, business and management, decision sciences and health professions.

**Methods:**

The structured literature review with its reliable and replicable research protocol allowed the researchers to extract 288 peer-reviewed papers from Scopus. The authors used qualitative and quantitative variables to analyse authors, journals, keywords, and collaboration networks among researchers. Additionally, the paper benefited from the Bibliometrix R software package.

**Results:**

The investigation showed that the literature in this field is emerging. It focuses on health services management, predictive medicine, patient data and diagnostics, and clinical decision-making. The United States, China, and the United Kingdom contributed the highest number of studies. Keyword analysis revealed that AI can support physicians in making a diagnosis, predicting the spread of diseases and customising treatment paths.

**Conclusions:**

The literature reveals several AI applications for health services and a stream of research that has not fully been covered. For instance, AI projects require skills and data quality awareness for data-intensive analysis and knowledge-based management. Insights can help researchers and health professionals understand and address future research on AI in the healthcare field.

## Background

Artificial intelligence (AI) generally applies to computational technologies that emulate mechanisms assisted by human intelligence, such as thought, deep learning, adaptation, engagement, and sensory understanding [1, 2]. Some devices can execute a role that typically involves human interpretation and decision-making [3, 4]. These techniques have an interdisciplinary approach and can be applied to different fields, such as medicine and health. AI has been involved in medicine since as early as the 1950s, when physicians made the first attempts to improve their diagnoses using computer-aided programs [5, 6]. Interest and advances in medical AI applications have surged in recent years due to the substantially enhanced computing power of modern computers and the vast amount of digital data available for collection and utilisation [7]. AI is gradually changing medical practice. There are several AI applications in medicine that can be used in a variety of medical fields, such as clinical, diagnostic, rehabilitative, surgical, and predictive practices. Another critical area of medicine where AI is making an impact is clinical decision-making and disease diagnosis. AI technologies can ingest, analyse, and report large volumes of data across different modalities to detect disease and guide clinical decisions [3, 8]. AI applications can deal with the vast amount of data produced in medicine and find new information that would otherwise remain hidden in the mass of medical big data [9–11]. These technologies can also identify new drugs for health services management and patient care treatments [5, 6].

Courage in the application of AI is visible through a search in the primary research databases. However, as Meskò et al. [7] find, the technology will potentially reduce care costs and repetitive operations by focusing the medical profession on critical thinking and clinical creativity. As Cho et al. and Doyle et al. [8, 9] add, the AI perspective is exciting; however, new studies will be needed to establish the efficacy and applications of AI in the medical field [10].

Our paper will also concentrate on AI strategies for healthcare from the accounting, business, and management perspectives. The authors used the structured literature review (SLR) method for its reliable and replicable research protocol [11] and selected bibliometric variables as sources of investigation. Bibliometric usage enables the recognition of the main quantitative variables of the study stream [12]. This method facilitates the detection of the required details of a particular research subject, including field authors, number of publications, keywords for interaction between variables (policies, properties and governance) and country data [13]. It also allows the application of the science mapping technique [14]. Our paper adopted the Bibliometrix R package and the biblioshiny web interface as tools of analysis [14].

The investigation offers the following insights for future researchers and practitioners:

- bibliometric information on 288 peer-reviewed English papers from the Scopus collection.
- Identification of leading journals in this field, such as *Journal of Medical Systems, Studies in Health Technology and Informatics, IEEE Journal of Biomedical and Health Informatics,* and *Decision Support Systems.*
- Qualitative and quantitative information on authors’ Lotka’s law, h-index, g-index, m-index, keyword, and citation data.
- Research on specific countries to assess AI in the delivery and effectiveness of healthcare, quotes, and networks within each region.
- A topic dendrogram study that identifies five research clusters: health services management, predictive medicine, patient data, diagnostics, and finally, clinical decision-making.
- An in-depth discussion that develops theoretical and practical implications for future studies.

The paper is organised as follows. Section 2 lists the main bibliometric articles in this field. Section 3 elaborates on the methodology. Section 4 presents the findings of the bibliometric analysis. Section 5 discusses the main elements of AI in healthcare based on the study results. Section 6 concludes the article with future implications for research.

## Related works and originality

As suggested by Zupic and Čater [15], a research stream can be evaluated with bibliometric methods that can introduce objectivity and mitigate researcher bias. For this reason, bibliometric methods are attracting increasing interest among researchers as a reliable and impersonal research analytical approach [16, 17]. Recently, bibliometrics has been an essential method for analysing and predicting research trends [18]. Table 1 lists other research that has used a similar approach in the research stream investigated.

**Table 1:** List of research using bibliometric analysis. *Source*: Authors’ elaboration

References	Field
Huang et al. [1]	Rehabilitation medicine
Hao et al. [2]	Text mining in medical research
	Medical big data
Liao et al. [3]	Global evolution of research on AI in health and medicine
dos Santos et al. [4]	Data mining and machine learning techniques applied to public health problems
Connelly et al. [5]	Robotic surgery
Guo et al. [6]	AI-related research conducted in the field of health problems
Choudhury et al. [7]	Machine learning in geriatric clinical
Choudhury and Asan [8]	AI in patient safety outcomes
This paper	AI techniques for clinical decision-making and data management quality in healthcare

The scientific articles reported show substantial differences in keywords and research topics that have been previously studied. The bibliometric analysis of Huang et al. [19] describes rehabilitative medicine using virtual reality technology. According to the authors, the primary goal of rehabilitation is to enhance and restore functional ability and quality of life for patients with physical impairments or disabilities. In recent years, many healthcare disciplines have been privileged to access various technologies that provide tools for both research and clinical intervention.

Hao et al. [20] focus on text mining in medical research. As reported, text mining reveals new, previously unknown information by using a computer to automatically extract information from different text resources. Text mining methods can be regarded as an extension of data mining to text data. Text mining is playing an increasingly significant role in processing medical information. Similarly, the studies by dos Santos et al. [21] focus on applying data mining and machine learning (ML) techniques to public health problems. As stated in this research, public health may be defined as the art and science of preventing diseases, promoting health, and prolonging life. Using data mining and ML techniques, it is possible to discover new information that otherwise would be hidden. These two studies are related to another topic: medical big data. According to Liao et al. [22], big data is a typical “buzzword” in the business and research community, referring to a great mass of digital data collected from various sources. In the medical field, we can obtain a vast amount of data (i.e., medical big data). Data mining and ML techniques can help deal with this information and provide helpful insights for physicians and patients. More recently, Choudhury et al. [23] provide a systematic review on the use of ML to improve the care of elderly patients, demonstrating eligible studies primarily in psychological disorders and eye diseases.

Tran et al. [2] focus on the global evolution of AI research in medicine. Their bibliometric analysis highlights trends and topics related to AI applications and techniques. As stated in Connelly et al.’s [24] study, robot-assisted surgeries have rapidly increased in recent years. Their bibliometric analysis demonstrates how robotic-assisted surgery has gained acceptance in different medical fields, such as urological, colorectal, cardiothoracic, orthopaedic, maxillofacial and neurosurgery applications. Additionally, the bibliometric analysis of Guo et al. [25] provides an in-depth study of AI publications through December 2019. The paper focuses on tangible AI health applications, giving researchers an idea of how algorithms can help doctors and nurses. A new stream of research related to AI is also emerging. In this sense, Choudhury and Asan’s [26] scientific contribution provides a systematic review of the AI literature to identify health risks for patients. They report on 53 studies involving technology for clinical alerts, clinical reports, and drug safety. Considering the considerable interest within this research stream, this analysis differs from the current literature for several reasons. It aims to provide in-depth discussion, considering mainly the business, management, and accounting fields and not dealing only with medical and health profession publications.

Additionally, our analysis aims to provide a bibliometric analysis of variables such as authors, countries, citations and keywords to guide future research perspectives for researchers and practitioners, as similar analyses have done for several publications in other research streams [15, 16, 27]. In doing so, we use a different database, Scopus, that is typically adopted in social sciences fields. Finally, our analysis will propose and discuss a dominant framework of variables in this field, and our analysis will not be limited to AI application descriptions.

## Methodology

This paper evaluated AI in healthcare research streams using the SLR method [11]. As suggested by Massaro et al. [11], an SLR enables the study of the scientific corpus of a research field, including the scientific rigour, reliability and replicability of operations carried out by researchers. As suggested by many scholars, the methodology allows qualitative and quantitative variables to highlight the best authors, journals and keywords and combine a systematic literature review and bibliometric analysis [27–30]. Despite its widespread use in business and management [16, 31], the SLR is also used in the health sector based on the same philosophy through which it was originally conceived [32, 33]. A methodological analysis of previously published articles reveals that the most frequently used steps are as follows [28, 31, 34]:

1. defining research questions;
2. writing the research protocol;
3. defining the research sample to be analysed;
4. developing codes for analysis; and
5. critically analysing, discussing, and identifying a future research agenda.

Considering the above premises, the authors believe that an SLR is the best method because it combines scientific validity, replicability of the research protocol and connection between multiple inputs.

As stated by the methodological paper, the first step is research question identification. For this purpose, we benefit from the analysis of Zupic and Čater [15], who provide several research questions for future researchers to link the study of authors, journals, keywords and citations. Therefore, RQ1 is “What are the most prominent authors, journal keywords and citations in the field of the research study?” Additionally, as suggested by Haleem et al. [35], new technologies, including AI, are changing the medical field in unexpected timeframes, requiring studies in multiple areas. Therefore, RQ2 is “How does artificial intelligence relate to healthcare, and what is the focus of the literature?” Then, as discussed by Massaro et al. [36], RQ3 is “What are the research applications of artificial intelligence for healthcare?”.

The first research question aims to define the qualitative and quantitative variables of the knowledge flow under investigation. The second research question seeks to determine the state of the art and applications of AI in healthcare. Finally, the third research question aims to help researchers identify practical and theoretical implications and future research ideas in this field.

**Fig. 1:**
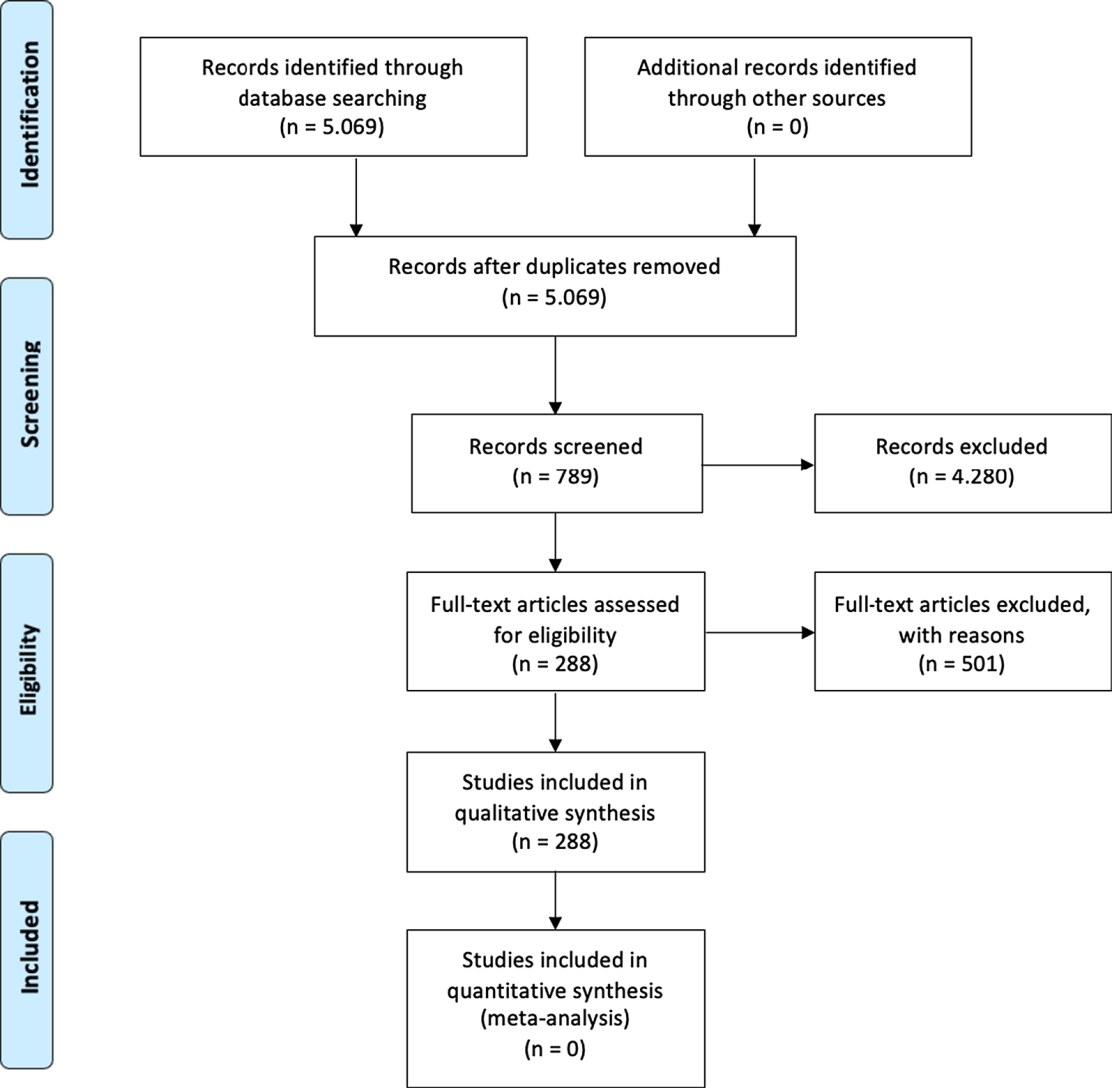
PRISMA workflow. *Source*: Authors’ elaboration on Liberati et al. [37]

The second fundamental step of the SLR is writing the research protocol [11]. Table 2 indicates the currently known literature elements, uniquely identifying the research focus, motivations and research strategy adopted and the results providing a link with the following points. Additionally, to strengthen the analysis, our investigation benefits from the PRISMA statement methodological article [37]. Although the SLR is a validated method for systematic reviews and meta-analyses, we believe that the workflow provided may benefit the replicability of the results [37–40]. Figure 1 summarises the researchers’ research steps, indicating that there are no results that can be referred to as a meta-analysis.

**Table 2:** SLR protocol and results obtained from Scopus. *Source*: Authors’ elaboration

Review protocol elements	Authors’ consideration	Results in terms of sources
What is already known? and Research topics	Artificial Intelligence (AI) is a novel topic based on already known knowledge, which has several healthcare implications. Due to its continuous growth, there is the potential for a structured literature review (SLR) investigating how AI can contribute to healthcare implementation	5069 results
Journals’ and thematic limitations	We have decided not to limit the research to an individual scientific journal because of the still young scope, as our analysis period. Therefore, our analysis is broad in terms of themes and period under investigation. It considers the following research streams: accounting, business, and management; decision sciences; health professions	789 results
Other restrictive elements	Authors selected only peer-reviewed articles and reviews, excluding conference proceedings, books and books chapters. Finally, only sources written in English are considered	288 results
Period of analysis	January 2021	-

The third step is to specify the search strategy and search database. Our analysis is based on the search string “Artificial Intelligence” OR “AI” AND “Healthcare” with a focus on “Business, Management, and Accounting”, “Decision Sciences”, and “Health professions”. As suggested by [11, 41] and motivated by [42], keywords can be selected through a top-down approach by identifying a large search field and then focusing on particular sub-topics. The paper uses data retrieved from the Scopus database, a multi-disciplinary database, which allowed the researchers to identify critical articles for scientific analysis [43]. Additionally, Scopus was selected based on Guo et al.’s [25] limitations, which suggest that “future studies will apply other databases, such as Scopus, to explore more potential papers”*.* The research focuses on articles and reviews published in peer-reviewed journals for their scientific relevance [11, 16, 17, 29] and does not include the grey literature, conference proceedings or books/book chapters. Articles written in any language other than English were excluded [2]. For transparency and replicability, the analysis was conducted on 11 January 2021. Using this research strategy, the authors retrieved 288 articles. To strengthen the study's reliability, we publicly provide the full bibliometric extract on the Zenodo repository [44, 45].

The fourth research phase is defining the code framework that initiates the analysis of the variables. The study will identify the following:

1. descriptive information of the research area;
2. source analysis [16];
3. author and citation analysis [28];
4. keywords and network analysis [14]; and
5. geographic distribution of the papers [14].

The final research phase is the article’s discussion and conclusion, where implications and future research trends will be identified.

At the research team level, the information is analysed with the statistical software R-Studio and the Bibliometrix package [15], which allows scientific analysis of the results obtained through the multi-disciplinary database.

## Results

The analysis of bibliometric results starts with a description of the main bibliometric statistics with the aim of answering RQ1, What are the most prominent authors, journal keywords and citations in the field of the research study?, and RQ2, How does artificial intelligence relate to healthcare, and what is the focus of the literature? Therefore, the following elements were thoroughly analysed: (1) type of document; (2) annual scientific production; (3) scientific sources; (4) source growth; (5) number of articles per author; (6) author’s dominance ranking; (7) author’s h-index, g-index, and m-index; (8) author’s productivity; (9) author’s keywords; (10) topic dendrogram; (11) a factorial map of the document with the highest contributions; (12) article citations; (13) country production; (14) country citations; (15) country collaboration map; and (16) country collaboration network.

### Main information

**Fig. 2:**
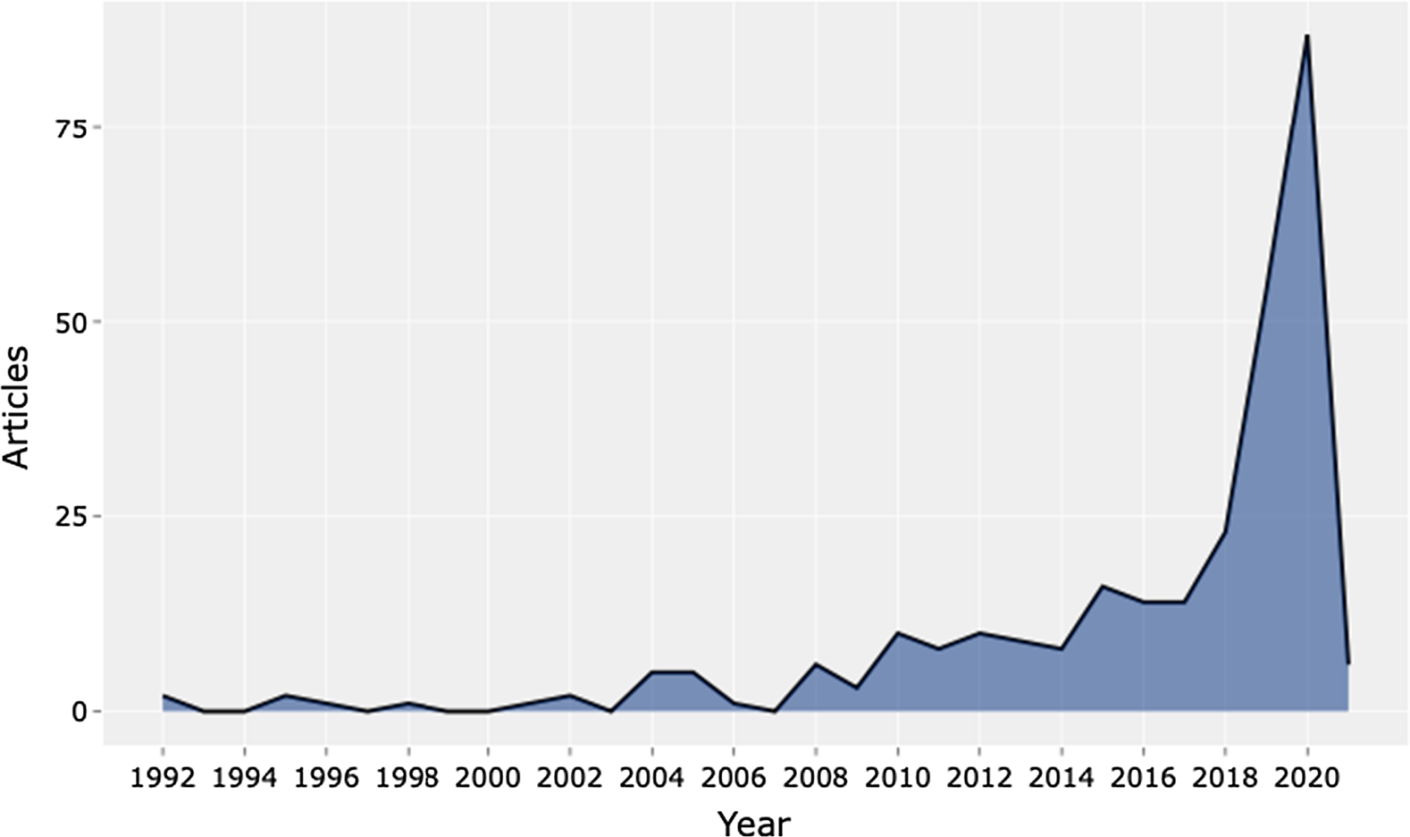
Annual scientific production. *Source*: Authors’ elaboration

Table 3 shows the information on 288 peer-reviewed articles published between 1992 and January 2021 extracted from the Scopus database. The number of keywords is 946 from 136 sources, and the number of keywords plus, referring to the number of keywords that frequently appear in an article’s title, was 2329. The analysis period covered 28 years and 1 month of scientific production and included an annual growth rate of 5.12%. However, the most significant increase in published articles occurred in the past three years (please see Fig. 2). On average, each article was written by three authors (3.56). Finally, the collaboration index (CI), which was calculated as the total number of authors of multi-authored articles/total number of multi-authored articles, was 3.97 [46].

**Table 3:** Main information. *Source*: Authors’ elaboration

Main information	Explanation	No
Documents	Total number of scientific papers and review	288
Sources	The frequency distribution of sources as journals	136
Author’s keywords	Total number of keywords	946
Keywords plus (ID)	Total number of phrases that frequently appear in the title of an article's references	2329
Period	Years of publication	1992–2021
Authors	Total number of authors	1025
Authors appearances	The authors’ frequency distribution	1059
Authors of single-authored documents	The number of single authors per articles	36
Authors of multi-authored documents	The number of authors of multi-authored articles	989
Authors per document	The average number of authors in each document	3.56
Co-authors per documents	The average number of co-authors in each document	3.68
Average citations per article	The average number of quotes in each article	12.68
Collaboration index (CI)		3.97

Table 4 shows the top 20 sources related to the topic. The Journal of Medical Systems is the most relevant source, with twenty-one of the published articles. This journal's main issues are the foundations, functionality, interfaces, implementation, impacts, and evaluation of medical technologies. Another relevant source is Studies in Health Technology and Informatics, with eleven articles. This journal aims to extend scientific knowledge related to biomedical technologies and medical informatics research. Both journals deal with cloud computing, machine learning, and AI as a disruptive healthcare paradigm based on recent publications. The IEEE Journal of Biomedical and Health Informatics investigates technologies in health care, life sciences, and biomedicine applications from a broad perspective. The next journal, Decision Support Systems, aims to analyse how these technologies support decision-making from a multi-disciplinary view, considering business and management. Therefore, the analysis of the journals revealed that we are dealing with an interdisciplinary research field. This conclusion is confirmed, for example, by the presence of purely medical journals, journals dedicated to the technological growth of healthcare, and journals with a long-term perspective such as futures.

**Table 4:** Main twenty sources. *Source*: Authors’ elaboration

Top 20 sources	No of articles
Journal of Medical Systems	21
Studies in Health Technology and Informatics	18
IEEE Journal of Biomedical and Health Informatics	17
Decision Support Systems	11
Healthcare Informatics Research	11
International Journal of Scientific and Technology Research	8
International Journal of Recent Technology and Engineering	7
Journal of Digital Imaging	7
NPJ Digital Medicine	5
Physiotherapy Theory and Practice	5
Telemedicine and E-Health	5
Biomedical Engineering Online	4
Information Sciences	4
International Journal of Healthcare Information Systems and Informatics	4
BMJ Health and Care Informatics	3
Futures	3
International Journal on Emerging Technologies	3
Journal of the Operational Research Society	3
Judgement and Decision Making	3
Medical Image Analysis	3

**Fig. 3:**
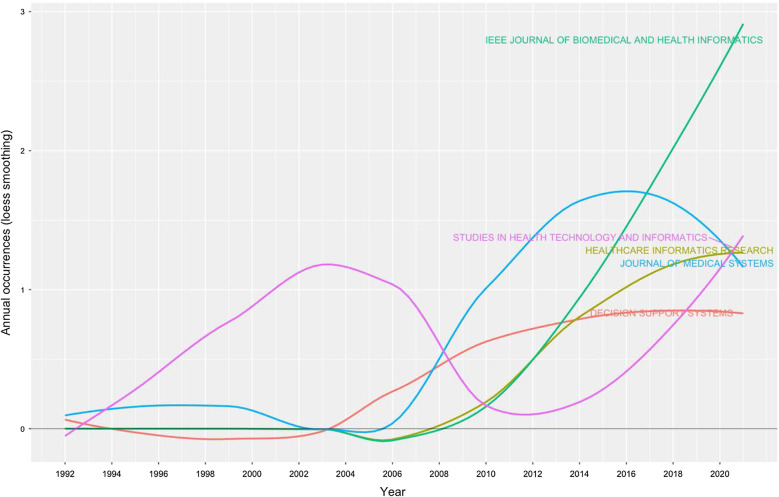
Source growth. *Source*: Authors’ elaboration

The distribution frequency of the articles (Fig. 3) indicates the journals dealing with the topic and related issues. Between 2008 and 2012, a significant growth in the number of publications on the subject is noticeable. However, the graph shows the results of the Loess regression, which includes the quantity and publication time of the journal under analysis as variables. This method allows the function to assume an unlimited distribution; that is, feature can consider values below zero if the data are close to zero. It contributes to a better visual result and highlights the discontinuity in the publication periods [47].

**Fig. 4:**
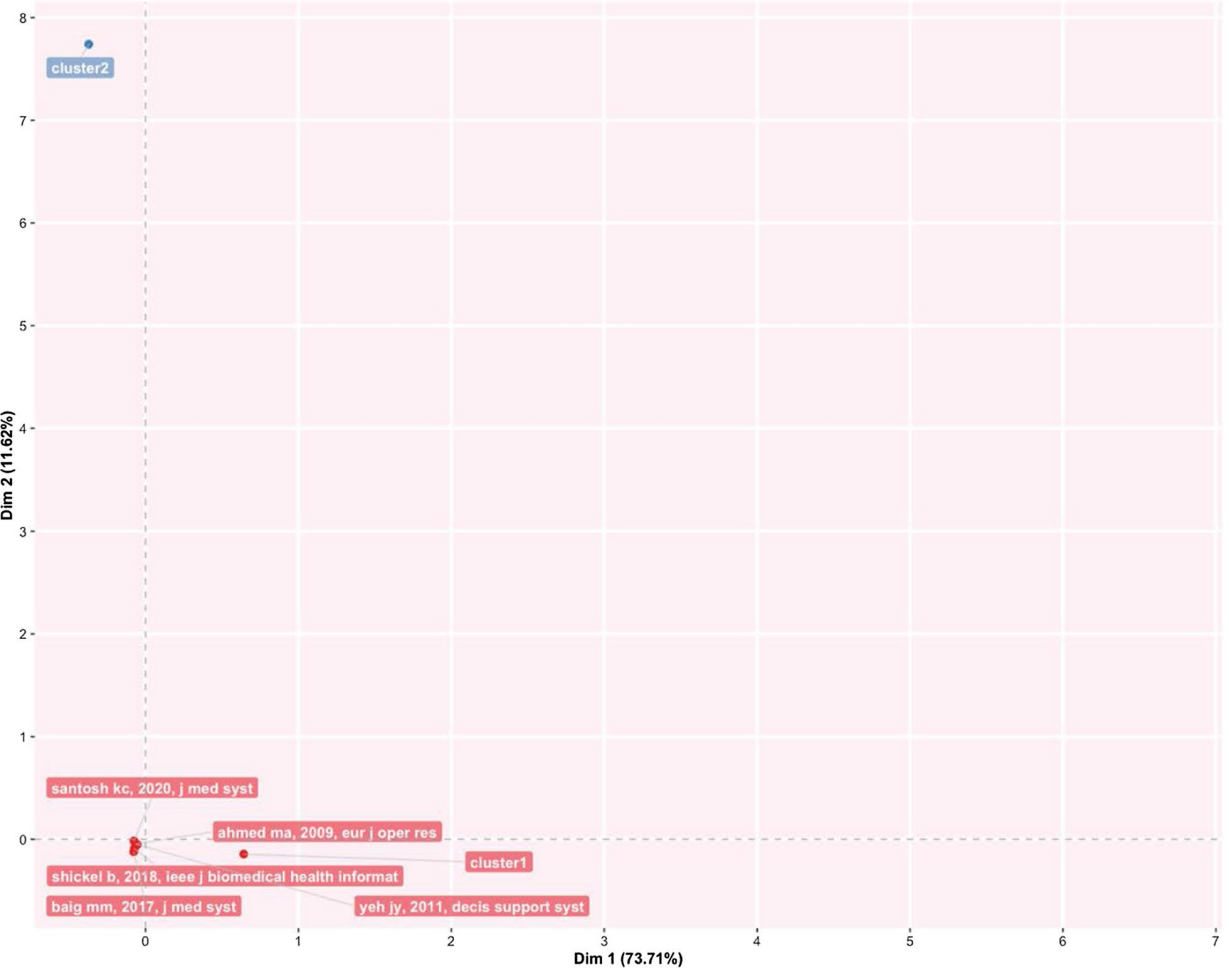
Factorial map of the most cited documents. *Source*: Authors’ elaboration

Finally, Fig. 4 provides an analytical perspective on factor analysis for the most cited papers. As indicated in the literature [48, 49], using factor analysis to discover the most cited papers allows for a better understanding of the scientific world’s intellectual structure. For example, our research makes it possible to consider certain publications that effectively analyse subject specialisation. For instance, Santosh’s [50] article addresses the new paradigm of AI with ML algorithms for data analysis and decision support in the COVID-19 period, setting a benchmark in terms of citations by researchers. Moving on to the application, an article by Shickel et al. [51] begins with the belief that the healthcare world currently has much health and administrative data. In this context, AI and deep learning will support medical and administrative staff in extracting data, predicting outcomes, and learning medical representations. Finally, in the same line of research, Baig et al. [52], with a focus on wearable patient monitoring systems (WPMs), conclude that AI and deep learning may be landmarks for continuous patient monitoring and support for healthcare delivery.

### Authors

This section identifies the most cited authors of articles on AI in healthcare. It also identifies the authors’ keywords, dominance factor (DF) ranking, h-index, productivity, and total number of citations. Table 5 identifies the authors and their publications in the top 20 rankings. As the table shows, Bushko R.G. has the highest number of publications: four papers. He is the editor-in-chief of Future of Health Technology, a scientific journal that aims to develop a clear vision of the future of health technology. Then, several authors each wrote three papers. For instance, Liu C. is a researcher active in the topic of ML and computer vision, and Sharma A. from Emory University Atlanta in the USA is a researcher with a clear focus on imaging and translational informatics. Some other authors have two publications each. While some authors have published as primary authors, most have published as co-authors. Hence, in the next section, we measure the contributory power of each author by investigating the DF ranking through the number of elements.

**Table 5:** Most cited authors. *Source*: Authors’ elaboration

Number of articles	Authors (top 20)
4	Bushko, RG
3	Liu C Sharma, A
2	Attia, S
	Beckstead, JW
	Bonezzi, A
	Boubetra, A
	Chang, H
	Chang, V
	Clifton, DA
	Das, S
	Fan, W
	Fox, J
	Kalhori, SRN
	Khan, O
	Liu, J
	Longoni, C
	Luo, G
	Michalowski, W
	Morewedge, CK

#### Authors’ dominance ranking

The dominance factor (DF) is a ratio measuring the fraction of multi-authored articles in which an author acts as the first author [53]. Several bibliometric studies use the DF in their analyses [46, 54]. The DF ranking calculates an author’s dominance in producing articles. The DF is calculated by dividing the number of an author’s multi-authored papers as the first author (Nmf) by the author's total number of multi-authored papers (Nmt). This is omitted in the single-author case due to the constant value of 1 for single-authored articles. This formulation could lead to some distortions in the results, especially in fields where the first author is entered by surname alphabetical order [55].

The mathematical equation for the DF is shown as:

$$DF \, = \frac{Nmf}{{Nmt}}$$

Table 6 lists the top 20 DF rankings. The data in the table show a low level of articles per author, either for first-authored or multi-authored articles. The results demonstrate that we are dealing with an emerging topic in the literature. Additionally, as shown in the table, Fox J. and Longoni C. are the most dominant authors in the field.

**Table 6:** Authors dominance factor. *Source*: Authors’ elaboration

Rank by DF	Author	Dominance factor	Total articles	Multi-authored	First-authored	Rank by articles
1	Fox, J	1.0000	2	2	2	2
1	Longoni, C	1.0000	2	2	2	2
1	Luo, G	1.0000	2	1	1	2
1	Pezzo, MV	1.0000	2	2	2	2
1	Saoud, MS	1.0000	2	2	2	2
1	Abdel-Basset, M	1.0000	1	1	1	14
1	Abderrahman, B	1.0000	1	1	1	14
1	Abhari, S	1.0000	1	1	1	14
1	Achunair, A	1.0000	1	1	1	14
1	Adde, L	1.0000	1	1	1	14
1	Aggarwal, L	1.0000	1	1	1	14
1	Clifton, DA	0.5000	2	2	1	2
13	Das, S	0.5000	2	2	1	2
13	Fan, W	0.5000	2	2	1	2
13	Liu, J	0.5000	2	2	1	2
13	Sonnessa, M	0.5000	2	2	1	2
13	Young, A	0.5000	2	2	1	2
13	Zhang, J	0.5000	2	2	1	2
20	Sharma, A	0.3333	3	3	1	1

#### Authors’ impact

Table 7 shows the impact of authors in terms of the h-index [56] (i.e., the productivity and impact of citations of a researcher), g-index [57] (i.e., the distribution of citations received by a researcher's publications), m-index [58] (i.e., the h-index value per year), total citations, total paper and years of scientific publication. The H-index was introduced in the literature as a metric for the objective comparison of scientific results and depended on the number of publications and their impact [59]. The results show that the 20 most relevant authors have an h-index between 2 and 1. For the practical interpretation of the data, the authors considered data published by the London School of Economics [60]. In the social sciences, the analysis shows values of 7.6 for economic publications by professors and researchers who had been active for several years. Therefore, the youthfulness of the research area has attracted young researchers and professors. At the same time, new indicators have emerged over the years to diversify the logic of the h-index. For example, the g-index indicates an author's impact on citations, considering that a single article can generate these. The m-index, on the other hand, shows the cumulative value over the years.

**Table 7:** Authors impact. *Source*: Authors’ elaboration

Author	h-index	g-index	m-index	Total citations	Total papers	Year Start
Bushko RG	1	2	0.050	4	4	2002
Liu C	2	3	0.500	10	3	2018
Sharma A	1	1	0.167	1	3	2016
Attia S	2	2	0.333	7	2	2016
Beckstead JW	1	1	0.500	2	2	2020
Bonezzi A	2	2	0.667	46	2	2019
Boubetra A	2	2	0.333	7	2	2016
Chang H	1	2	0.200	8	2	2017
Chang V	1	2	0.250	14	2	2018
Clifton DA	1	2	0.111	20	2	2013
Das S	1	1	0.333	3	2	2019
Fan W	2	2	1.000	20	2	2020
Fox J	1	1	0.500	2	2	2020
Kalhori SRN	2	2	0.400	13	2	2017
Khan O	1	1	0.500	2	2	2020
Liu J	1	2	0.250	11	2	2018
Longoni C	2	2	0.667	46	2	2019
Luo G	2	2	0.200	20	2	2012
Michalowski W	2	2	0.154	14	2	2009
Morewedge CK	2	2	0.667	46	2	2019

The analysis, also considering the total number of citations, the number of papers published and the year of starting to publish, thus confirms that we are facing an expanding research flow.

#### Authors’ productivity

**Fig. 5:**
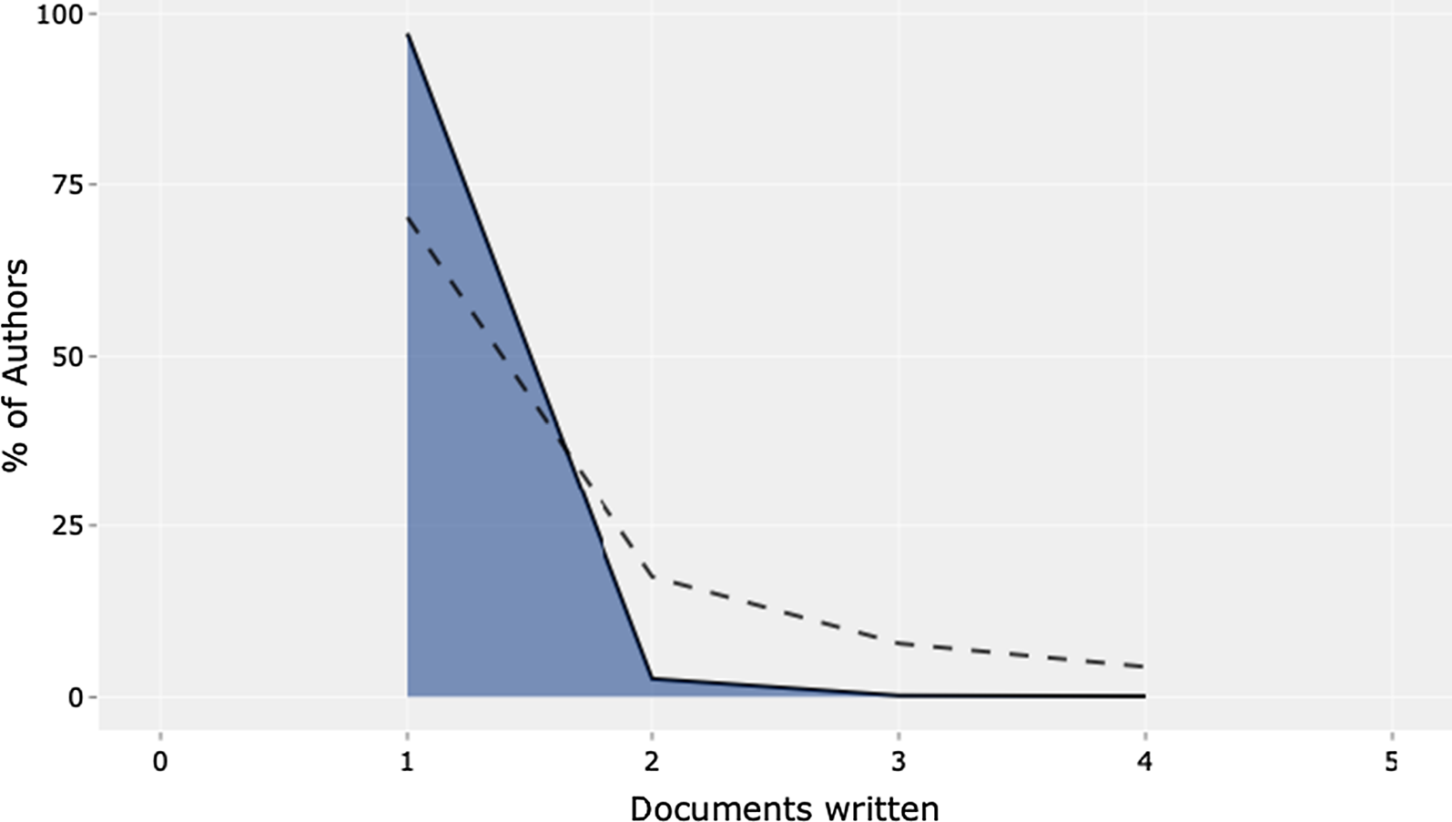
Lotka’s law. *Source*: Authors’ elaboration

Figure 5 shows Lotka’s law. This mathematical formulation originated in 1926 to describe the publication frequency by authors in a specific research field [61]. In practice, the law states that the number of authors contributing to research in a given period is a fraction of the number who make up a single contribution [14, 61].

The mathematical relationship is expressed in reverse in the following way:

$$x^{n } * y_{x} = C$$

where y_x_ is equal to the number of authors producing x articles in each research field. Therefore, C and n are constants that can be estimated in the calculation.

The figure's results are in line with Lotka's results, with an average of two publications per author in a given research field. In addition, the figure shows the percentage of authors. Our results lead us to state that we are dealing with a young and growing research field, even with this analysis. Approximately 70% of the authors had published only their first research article. Only approximately 20% had published two scientific papers.

#### Authors’ keywords

This section provides information on the relationship between the keywords *artificial intelligence* and *healthcare*. This analysis is essential to determine the research trend, identify gaps in the discussion on AI in healthcare, and identify the fields that can be interesting as research areas [42, 62].

Table 8 highlights the total number of keywords per author in the top 20 positions. The ranking is based on the following elements: *healthcare, artificial intelligence,* and *clinical decision support system*. Keyword analysis confirms the scientific area of reference. In particular, we deduce the definition as “Artificial intelligence is the theory and development of computer systems able to perform tasks normally requiring human intelligence, such as visual perception, speech recognition, decision-making, and translation between languages” [2, 63]. Panch et al. [4] find that these technologies can be used in different business and management areas. After the first keyword, the analysis reveals AI applications and related research such as machine learning and deep learning.

**Table 8:** Author’s keywords in articles on artificial intelligence in healthcare. *Source*: Authors’ elaboration

Author keywords (top 20)	Occurrences
Artificial intelligence	64
Machine learning	34
Healthcare	31
Deep learning	19
Data mining	10
Big data	9
Decision support system	9
Natural language processing	9
Data analytics	7
Decision support systems	7
Classification	6
COVID-19	6
Predictive analysis	6
Telemedicine	6
AI	5
Clinical decision support system	5
Descriptive analysis	5
Internet of things	5
Medical informatics	5
mHealth	5

Additionally, data mining and big data are a step forward in implementing exciting AI applications. According to our specific interest, if we applied AI in healthcare, we would achieve technological applications to help and support doctors and medical researchers in decision-making. The link between AI and decision-making is the reason why we find, in the seventh position, the keyword *clinical decision support system*. AI techniques can unlock clinically relevant information hidden in the massive amount of data that can assist clinical decision-making [64]. If we analyse the following keywords, we find other elements related to decision-making and support systems.

**Fig. 6:**
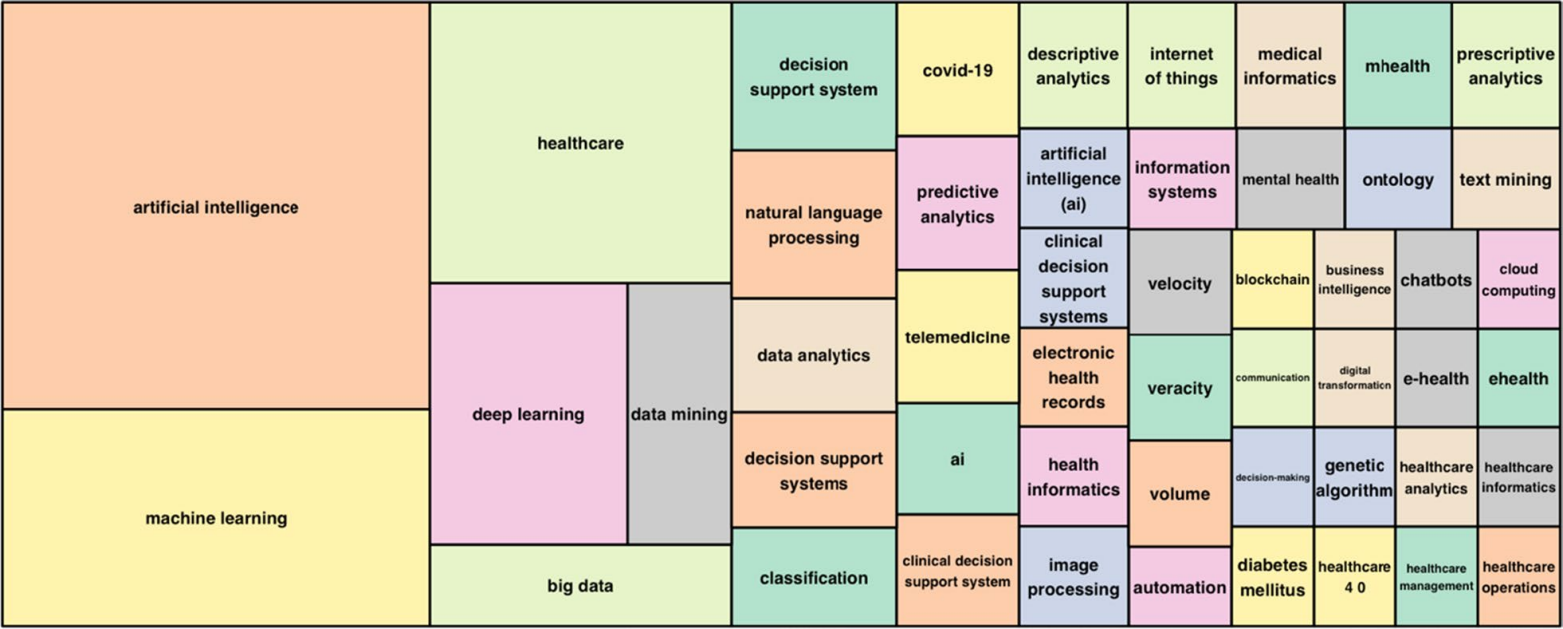
Keywords treemap. *Source*: Authors’ elaboration

The TreeMap below (Fig. 6) highlights the combination of possible keywords representing AI and healthcare.

**Fig. 7:**
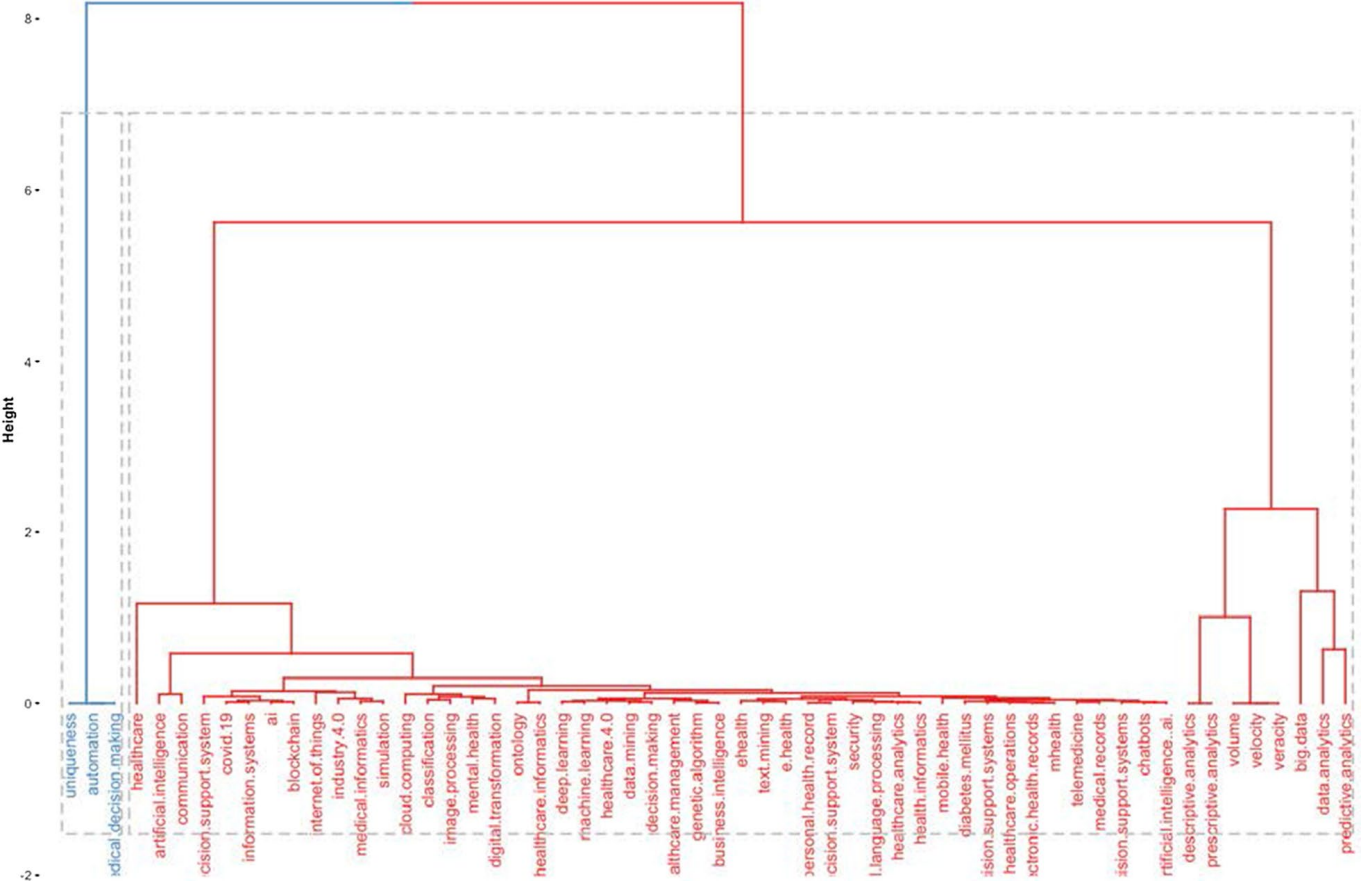
Topic dendrogram. *Source*: Authors’ elaboration

The topic dendrogram in Fig. 7 represents the hierarchical order and the relationship between the keywords generated by hierarchical clustering [42]. The cut in the figure and the vertical lines facilitate an investigation and interpretation of the different clusters. As stated by Andrews [48], the figure is not intended to find the perfect level of associations between clusters. However, it aims to estimate the approximate number of clusters to facilitate further discussion.

The research stream of AI in healthcare is divided into two main strands. The blue strand focuses on medical information systems and the internet. Some papers are related to healthcare organisations, such as the Internet of Things, meaning that healthcare organisations use AI to support health services management and data analysis. AI applications are also used to improve diagnostic and therapeutic accuracy and the overall clinical treatment process [2]. If we consider the second block, the red one, three different clusters highlight separate aspects of the topic. The first could be explained as AI and ML predictive algorithms. Through AI applications, it is possible to obtain a predictive approach that can ensure that patients are better monitored. This also allows a better understanding of risk perception for doctors and medical researchers. In the second cluster, the most frequent words are *decisions*, *information system*, and *support system*. This means that AI applications can support doctors and medical researchers in decision-making. Information coming from AI technologies can be used to consider difficult problems and support a more straightforward and rapid decision-making process. In the third cluster, it is vital to highlight that the ML model can deal with vast amounts of data. From those inputs, it can return outcomes that can optimise the work of healthcare organisations and scheduling of medical activities.

**Fig. 8:**
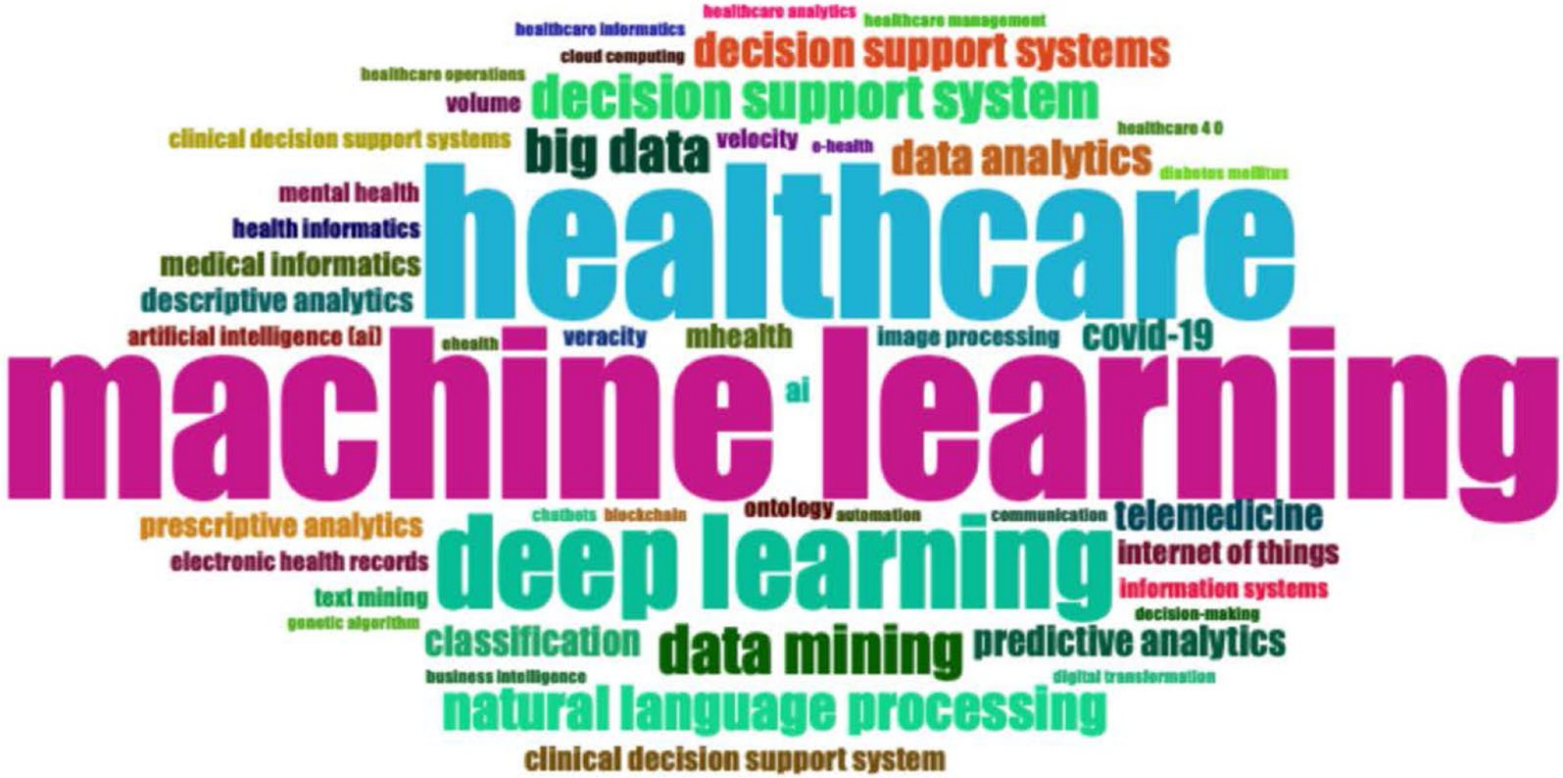
Word cloud. *Source*: Authors’ elaboration

Furthermore, the word cloud in Fig. 8 highlights aspects of AI in healthcare, such as decision support systems, decision-making, health services management, learning systems, ML techniques and diseases. The figure depicts how AI is linked to healthcare and how it is used in medicine.

**Fig. 9:**
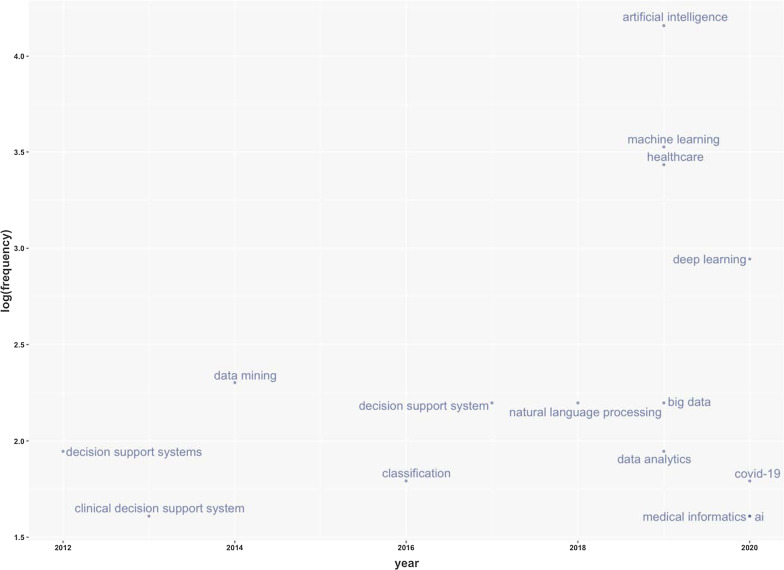
Keywords frequency. *Source*: Authors’ elaboration

Figure 9 represents the search trends based on the keywords analysed. The research started in 2012. First, it identified research topics related to clinical decision support systems. This topic was recurrent during the following years. Interestingly, in 2018, studies investigated AI and natural language processes as possible tools to manage patients and administrative elements. Finally, a new research stream considers AI's role in fighting COVID-19 [65, 66].

Table 9 represents the number of citations from other articles within the top 20 rankings. The analysis allows the benchmark studies in the field to be identified [48]. For instance, Burke et al. [67] writes the most cited paper and analyses efficient nurse rostering methodologies. The paper critically evaluates tangible interdisciplinary solutions that also include AI. Immediately thereafter, Ahmed M.A.'s article proposes a data-driven optimisation methodology to determine the optimal number of healthcare staff to optimise patients' productivity [68]. Finally, the third most cited article lays the groundwork for developing deep learning by considering diverse health and administrative information [51].

**Table 9:** Authors and sources citations. *Source*: Authors’ elaboration

Ranking no	Authors and their sources (top 20)	Total citations (number of citations received)	Total citation per year
1	Burke EK., 2014, J Scheduling	604	33.556
2	Ahmed MA., 2009, Eur J Oper Res	215	16.538
3	Shickel B., 2018, IEEE J Biomedical Health Informat	212	53,000
4	Liao Y., 2011, Morb Mortal Wkly Rep	149	13.544
5	Fusco C., 2011, Physiother Theory Pract	78	7.090
6	Baig MM., 2017, J Med Syst	76	15.200
7	Yeh JY., 2011, Decision Support Syst	76	6.909
8	Santosh KC., 2020, J Med Syst	72	36.000
9	Classen DC., 1992, Hosp Pharm	60	2.000
10	Mozaffari-Kermani M., 2015, IEEE J Biomedical Health Informat	58	8.286
11	Yan H., 2015, J Manag Anal	55	7.857
12	Longoni C., 2019, J Consum Res	44	14.667
13	Isern D., 2016, J Med Syst	44	7.333
14	Gayathri KS., 2015, Pers Ubiquitous Comp	44	6.286
15	Ben Ayed M., 2010, Decision Support Syst	44	3.667
16	Reiner B., 2010, J Digit Imaging	44	3.667
17	Li Y., 2016, Inf Sci	43	7.167
18	Rahulamathavan Y., 2014, IEEE J Biomedical Health Informat	41	5.125
19	Johnson MP., 2014, Decision Support System	37	4.625
20	Gmez-Vallejo HJ., 2016, Decision Support Syst	36	6.000

### Country

This section analyses the diffusion of AI in healthcare around the world. It highlights countries to show the geographies of this research. It includes all published articles, the total number of citations, and the collaboration network. The following sub-sections start with an analysis of the total number of published articles.

#### Country total articles

Figure 9 and Table 10 display the countries where AI in healthcare has been considered. The USA tops the list of countries with the maximum number of articles on the topic (215). It is followed by China (83), the UK (54), India (51), Australia (54), and Canada (32). It is immediately evident that the theme has developed on different continents, highlighting a growing interest in AI in healthcare. The figure shows that many areas, such as Russia, Eastern Europe and Africa except for Algeria, Egypt, and Morocco, have still not engaged in this scientific debate.

**Table 10:** Total number of articles per country. *Source*: Authors’ elaboration

Country	Total number of articles
USA	215
China	83
UK	54
India	51
Australia	34
Canada	32
South Korea	28
Spain	21
Italy	20
Germany	13
France	11
Iran	11
Turkey	11
Finland	9
Greece	9
Portugal	9
Netherlands	8
Norway	7

#### Country publications and collaboration map

**Fig. 10:**
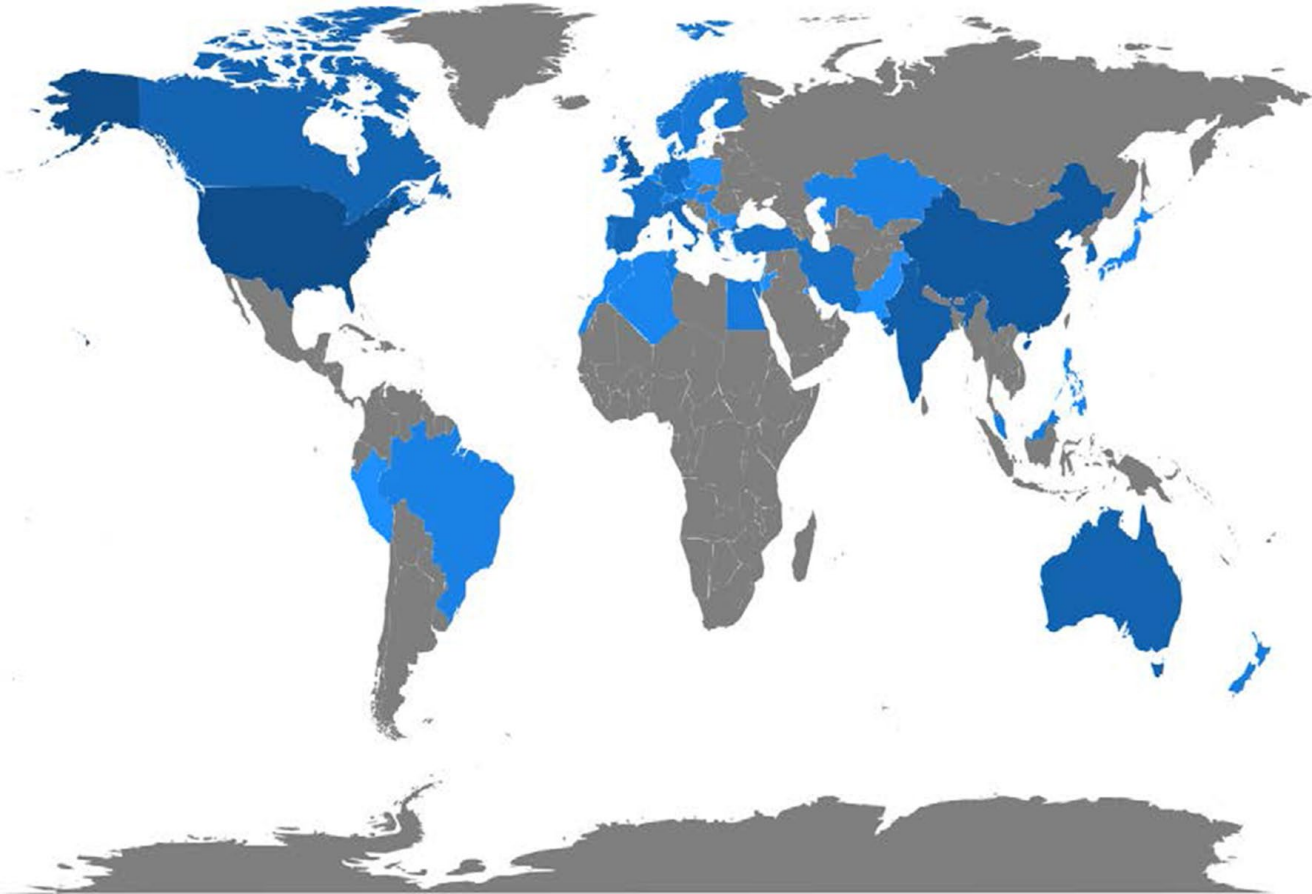
Articles per country. *Source*: Authors’ elaboration

This section discusses articles on AI in healthcare in terms of single or multiple publications in each country. It also aims to observe collaboration and networking between countries. Table 11 and Fig. 10 highlight the average citations by state and show that the UK, the USA, and Kuwait have a higher average number of citations than other countries. Italy, Spain and New Zealand have the most significant number of citations.

**Table 11:** Country and their total number of citations. *Source*: Authors’ elaboration

Country	Total citations	Average article citations
UK	680	97.14
USA	545	17.58
Kuwait	215	215.00
Italy	161	17.89
Spain	122	20.33
New Zealand	112	56.00
China	107	10.70
Korea	51	5.10
Tunisia	48	24.00
Australia	36	18.00
Canada	35	5.00
Hong Kong	31	7.75
Greece	30	30.00
Japan	29	29.00

**Fig. 11:**
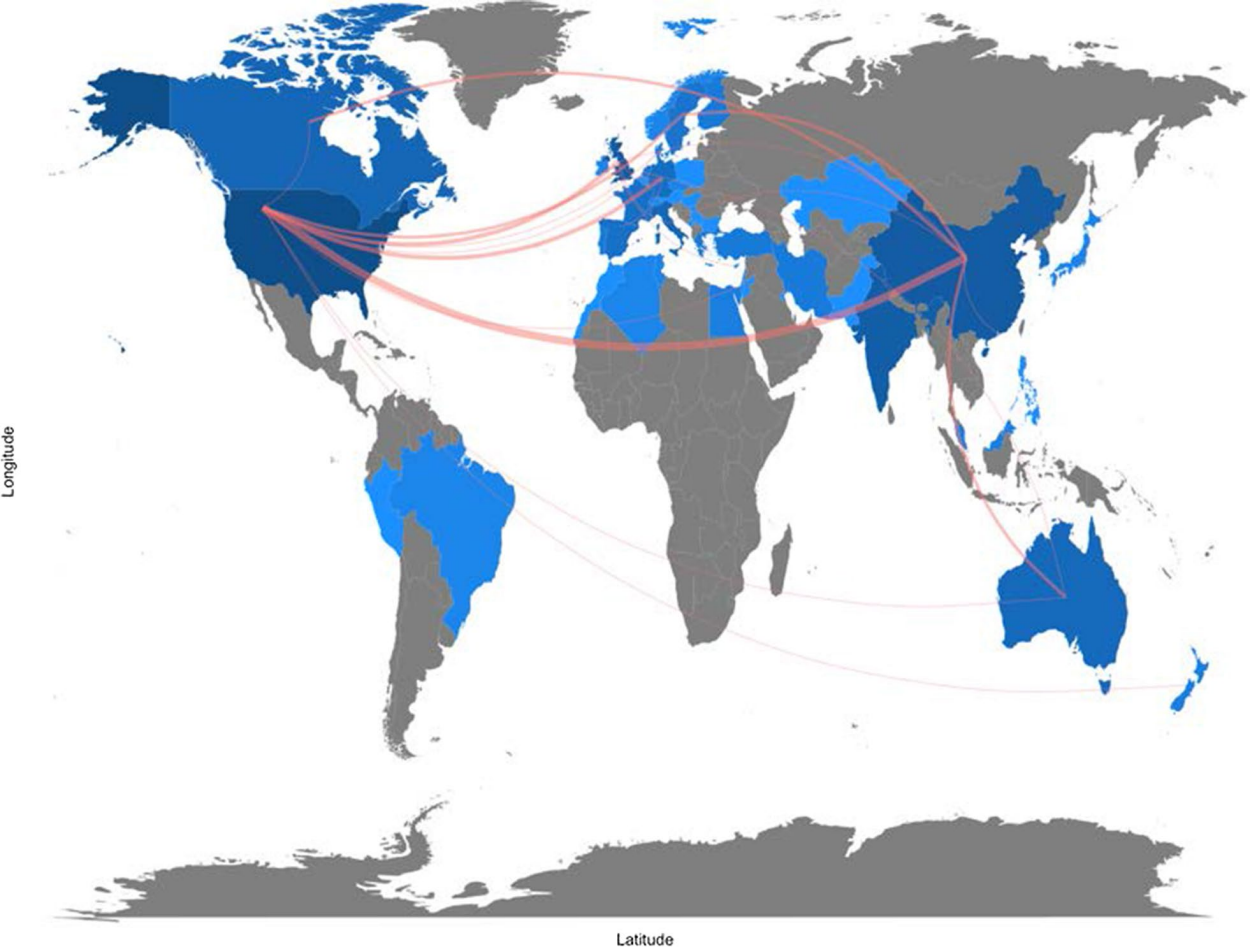
Collaboration map. *Source*: Authors’ elaboration

Figure 11 depicts global collaborations. The blue colour on the map represents research cooperation among nations. Additionally, the pink border linking states indicates the extent of collaboration between authors. The primary cooperation between nations is between the USA and China, with two collaborative articles. Other collaborations among nations are limited to a few papers.

### Artificial intelligence for healthcare: applications

This section aims to strengthen the research scope by answering RQ3: What are the research applications of artificial intelligence for healthcare?

**Fig. 12:**
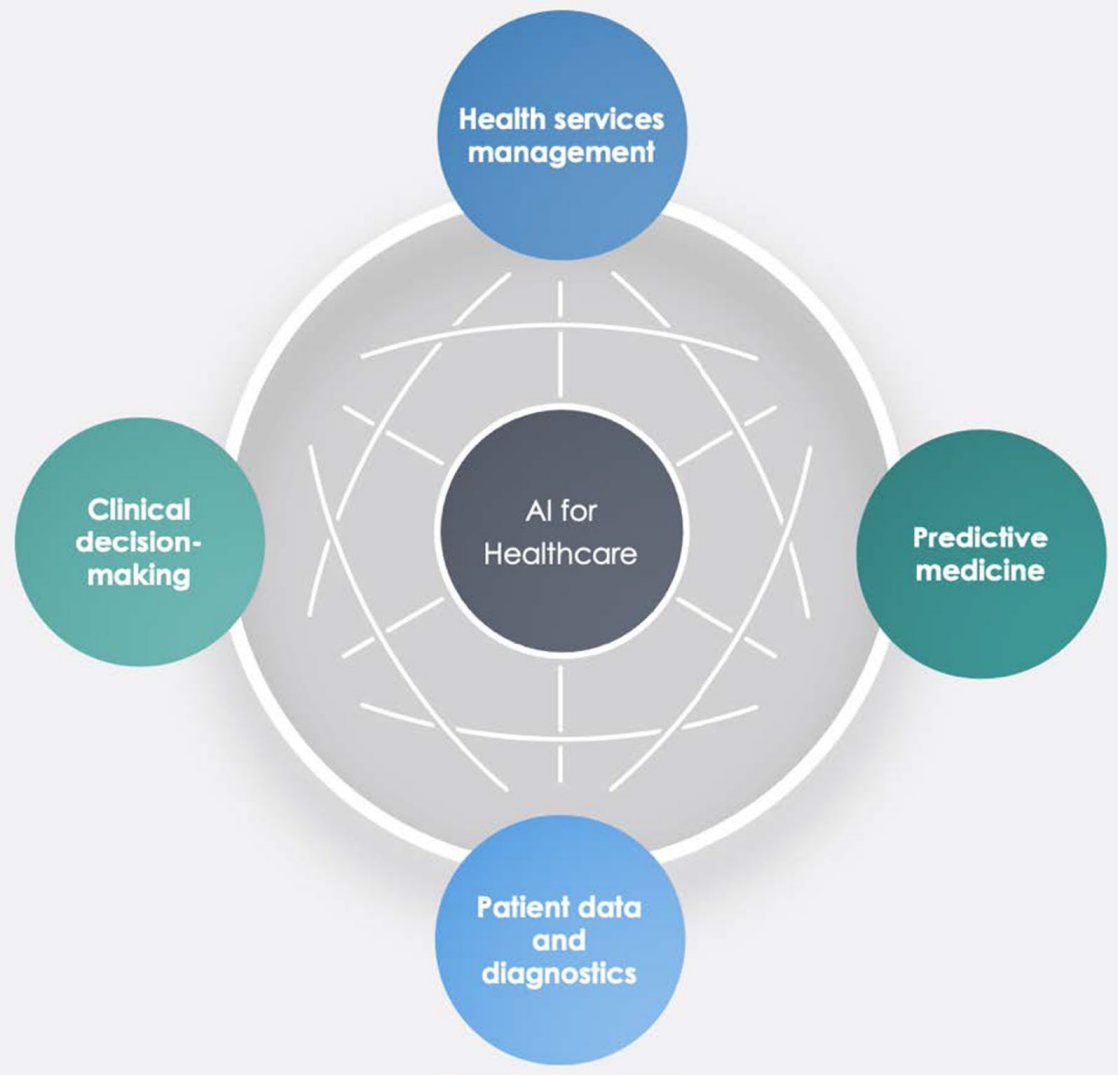
Dominant variables for AI in healthcare. *Source*: Authors’ elaboration

Benefiting from the topical dendrogram, researchers will provide a development model based on four relevant variables [69, 70]. AI has been a disruptive innovation in healthcare [4]. With its sophisticated algorithms and several applications, AI has assisted doctors and medical professionals in the domains of health information systems, geocoding health data, epidemic and syndromic surveillance, predictive modelling and decision support, and medical imaging [2, 9, 10, 64]. Furthermore, the researchers considered the bibliometric analysis to identify four macro-variables dominant in the field and used them as authors' keywords. Therefore, the following sub-sections aim to explain the debate on applications in healthcare for AI techniques. These elements are shown in Fig. 12.

#### Health services management

One of the notable aspects of AI techniques is potential support for comprehensive health services management. These applications can support doctors, nurses and administrators in their work. For instance, an AI system can provide health professionals with constant, possibly real-time medical information updates from various sources, including journals, textbooks, and clinical practices [2, 10]. These applications' strength is becoming even more critical in the COVID-19 period, during which information exchange is continually needed to properly manage the pandemic worldwide [71]. Other applications involve coordinating information tools for patients and enabling appropriate inferences for health risk alerts and health outcome prediction [72]. AI applications allow, for example, hospitals and all health services to work more efficiently for the following reasons:

- Clinicians can access data immediately when they need it.
- Nurses can ensure better patient safety while administering medication.
- Patients can stay informed and engaged in their care by communicating with their medical teams during hospital stays.

Additionally, AI can contribute to optimising logistics processes, for instance, realising drugs and equipment in a just-in-time supply system based totally on predictive algorithms [73, 74]. Interesting applications can also support the training of personnel working in health services. This evidence could be helpful in bridging the gap between urban and rural health services [75]. Finally, health services management could benefit from AI to leverage the multiplicity of data in electronic health records by predicting data heterogeneity across hospitals and outpatient clinics, checking for outliers, performing clinical tests on the data, unifying patient representation, improving future models that can predict diagnostic tests and analyses, and creating transparency with benchmark data for analysing services delivered [51, 76].

#### Predictive medicine

Another relevant topic is AI applications for disease prediction and diagnosis treatment, outcome prediction and prognosis evaluation [72, 77]. Because AI can identify meaningful relationships in raw data, it can support diagnostic, treatment and prediction outcomes in many medical situations [64]. It allows medical professionals to embrace the proactive management of disease onset. Additionally, predictions are possible for identifying risk factors and drivers for each patient to help target healthcare interventions for better outcomes [3]. AI techniques can also help design and develop new drugs, monitor patients and personalise patient treatment plans [78]. Doctors benefit from having more time and concise data to make better patient decisions. Automatic learning through AI could disrupt medicine, allowing prediction models to be created for drugs and exams that monitor patients over their whole lives [79].

#### Clinical decision-making

One of the keyword analysis main topics is that AI applications could support doctors and medical researchers in the clinical decision-making process. According to Jiang et al. [64], AI can help physicians make better clinical decisions or even replace human judgement in healthcare-specific functional areas. According to Bennett and Hauser [80], algorithms can benefit clinical decisions by accelerating the process and the amount of care provided, positively impacting the cost of health services. Therefore, AI technologies can support medical professionals in their activities and simplify their jobs [4]. Finally, as Redondo and Sandoval [81] find, algorithmic platforms can provide virtual assistance to help doctors understand the semantics of language and learning to solve business process queries as a human being would.

#### Patient data and diagnostics

Another challenging topic related to AI applications is patient data and diagnostics. AI techniques can help medical researchers deal with the vast amount of data from patients (i.e., *medical big data*). AI systems can manage data generated from clinical activities, such as screening, diagnosis, and treatment assignment. In this way, health personnel can learn similar subjects and associations between subject features and outcomes of interest [64].

These technologies can analyse raw data and provide helpful insights that can be used in patient treatments. They can help doctors in the diagnostic process; for example, to realise a high-speed body scan, it will be simpler to have an overall patient condition image. Then, AI technology can recreate a 3D mapping solution of a patient’s body.

In terms of data, interesting research perspectives are emerging. For instance, we observed the emergence of a stream of research on patient data management and protection related to AI applications [82].

For diagnostics, AI techniques can make a difference in rehabilitation therapy and surgery. Numerous robots have been designed to support and manage such tasks. Rehabilitation robots physically support and guide, for example, a patient’s limb during motor therapy [83]. For surgery, AI has a vast opportunity to transform surgical robotics through devices that can perform semi-automated surgical tasks with increasing efficiency. The final aim of this technology is to automate procedures to negate human error while maintaining a high level of accuracy and precision [84]. Finally, the -19 period has led to increased remote patient diagnostics through telemedicine that enables remote observation of patients and provides physicians and nurses with support tools [66, 85, 86].

## Discussion

This study aims to provide a bibliometric analysis of publications on AI in healthcare, focusing on accounting, business and management, decision sciences and health profession studies. Using the SLR method of Massaro et al. [11], we provide a reliable and replicable research protocol for future studies in this field. Additionally, we investigate the trend of scientific publications on the subject, unexplored information, future directions, and implications using the science mapping workflow. Our analysis provides interesting insights.

In terms of bibliometric variables, the four leading journals, *Journal of Medical Systems*, *Studies in Health Technology and Informatics*, *IEEE Journal of Biomedical and Health Informatics*, and *Decision Support Systems*, are optimal locations for the publication of scientific articles on this topic. These journals deal mainly with healthcare, medical information systems, and applications such as cloud computing, machine learning, and AI. Additionally, in terms of h-index, Bushko R.G. and Liu C. are the most productive and impactful authors in this research stream. Burke et al.’s [67] contribution is the most cited with an analysis of nurse rostering using new technologies such as AI. Finally, in terms of keywords, co-occurrence reveals some interesting insights. For instance, researchers have found that AI has a role in diagnostic accuracy and helps in the analysis of health data by comparing thousands of medical records, experiencing automatic learning with clinical alerts, efficient management of health services and places of care, and the possibility of reconstructing patient history using these data.

Second, this paper finds five cluster analyses in healthcare applications: health services management, predictive medicine, patient data, diagnostics, and finally, clinical decision-making. These technologies can also contribute to optimising logistics processes in health services and allowing a better allocation of resources.

Third, the authors analysing the research findings and the issues under discussion strongly support AI's role in decision support. These applications, however, are demonstrated by creating a direct link to data quality management and the technology awareness of health personnel [87].

### The importance of data quality for the decision-making process

Several authors have analysed AI in the healthcare research stream, but in this case, the authors focus on other literature that includes business and decision-making processes. In this regard, the analysis of the search flow reveals a double view of the literature. On the one hand, some contributions belong to the positivist literature and embrace future applications and implications of technology for health service management, data analysis and diagnostics [6, 80, 88]. On the other hand, some investigations also aim to understand the darker sides of technology and its impact. For example, as Carter [89] states, the impact of AI is multi-sectoral; its development, however, calls for action to protect personal data. Similarly, Davenport and Kalakota [77] focus on the ethical implications of using AI in healthcare. According to the authors, intelligent machines raise issues of accountability, transparency, and permission, especially in automated communication with patients. Our analysis does not indicate a marked strand of the literature; therefore, we argue that the discussion of elements such as the transparency of technology for patients is essential for the development of AI applications.

A large part of our results shows that, at the application level, AI can be used to improve medical support for patients (Fig. 11) [64, 82]. However, we believe that, as indicated by Kalis et al. [90] on the pages of Harvard Business Review, the management of costly back-office problems should also be addressed.

The potential of algorithms includes data analysis. There is an immense quantity of data accessible now, which carries the possibility of providing information about a wide variety of medical and healthcare activities [91]. With the advent of modern computational methods, computer learning and AI techniques, there are numerous possibilities [79, 83, 84]. For example, AI makes it easier to turn data into concrete and actionable observations to improve decision-making, deliver high-quality patient treatment, adapt to real-time emergencies, and save more lives on the clinical front. In addition, AI makes it easier to leverage capital to develop systems and facilities and reduce expenses at the organisational level [78]. Studying contributions to the topic, we noticed that data accuracy was included in the debate, indicating that a high standard of data will benefit decision-making practitioners [38, 77]. AI techniques are an essential instrument for studying data and the extraction of medical insight, and they may assist medical researchers in their practices. Using computational tools, healthcare stakeholders may leverage the power of data not only to evaluate past data (*descriptive analytics*) but also to forecast potential outcomes (*predictive analytics*) and to define the best actions for the present scenario (*prescriptive analytics*) [78]. The current abundance of evidence makes it easier to provide a broad view of patient health; doctors should have access to the correct details at the right time and location to provide the proper treatment [92].

### Will medical technology de-skill doctors?

Further reflection concerns the skills of doctors. Studies have shown that healthcare personnel are progressively being exposed to technology for different purposes, such as collecting patient records or diagnosis [71]. This is demonstrated by the keywords (Fig. 6) that focus on technology and the role of decision-making with new innovative tools. In addition, the discussion expands with Lu [93], which indicates that the excessive use of technology could hinder doctors’ skills and clinical procedures' expansion. Among the main issues arising from the literature is the possible de-skilling of healthcare staff due to reduced autonomy in decision-making concerning patients [94]. Therefore, the challenges and discussion we uncovered in Fig. 11 are expanded by also considering the ethical implications of technology and the role of skills.

### Implications

Our analysis also has multiple theoretical and practical implications.

In terms of theoretical contribution, this paper extends the previous results of Connelly et al., dos Santos et al, Hao et al., Huang et al., Liao et al. and Tran et al. [2, 19–22, 24] in considering AI in terms of clinical decision-making and data management quality.

In terms of practical implications, this paper aims to create a fruitful discussion with healthcare professionals and administrative staff on how AI can be at their service to increase work quality. Furthermore, this investigation offers a broad comprehension of bibliometric variables of AI techniques in healthcare. It can contribute to advancing scientific research in this field.

### Limitations

Like any other, our study has some limitations that could be addressed by more in-depth future studies. For example, using only one research database, such as Scopus, could be limiting. Further analysis could also investigate the PubMed, IEEE, and Web of Science databases individually and holistically, especially the health parts. Then, the use of search terms such as "Artificial Intelligence" OR "AI" AND "Healthcare" could be too general and exclude interesting studies. Moreover, although we analysed 288 peer-reviewed scientific papers, because the new research topic is new, the analysis of conference papers could return interesting results for future researchers. Additionally, as this is a young research area, the analysis will be subject to recurrent obsolescence as multiple new research investigations are published. Finally, although bibliometric analysis has limited the subjectivity of the analysis [15], the verification of recurring themes could lead to different results by indicating areas of significant interest not listed here.

### Future research avenues

Concerning future research perspectives, researchers believe that an analysis of the overall amount that a healthcare organisation should pay for AI technologies could be helpful. If these technologies are essential for health services management and patient treatment, governments should invest and contribute to healthcare organisations' modernisation. New investment funds could be made available in the healthcare world, as in the European case with the Next Generation EU programme or national investment programmes [95]. Additionally, this should happen especially in the poorest countries around the world, where there is a lack of infrastructure and services related to health and medicine [96]. On the other hand, it might be interesting to evaluate additional profits generated by healthcare organisations with AI technologies compared to those that do not use such technologies.

Further analysis could also identify why some parts of the world have not conducted studies in this area. It would be helpful to carry out a comparative analysis between countries active in this research field and countries that are not currently involved. It would make it possible to identify variables affecting AI technologies' presence or absence in healthcare organisations. The results of collaboration between countries also present future researchers with the challenge of greater exchanges between researchers and professionals. Therefore, further research could investigate the difference in vision between professionals and academics.

In the accounting, business, and management research area, there is currently a lack of quantitative analysis of the costs and profits generated by healthcare organisations that use AI technologies. Therefore, research in this direction could further increase our understanding of the topic and the number of healthcare organisations that can access technologies based on AI. Finally, as suggested in the discussion section, more interdisciplinary studies are needed to strengthen AI links with data quality management and AI and ethics considerations in healthcare.

## Conclusion

In pursuing the philosophy of Massaro et al.’s [11] methodological article, we have climbed on the shoulders of giants, hoping to provide a bird's-eye view of the AI literature in healthcare. We performed this study with a bibliometric analysis aimed at discovering authors, countries of publication and collaboration, and keywords and themes. We found a fast-growing, multi-disciplinary stream of research that is attracting an increasing number of authors.

The research, therefore, adopts a quantitative approach to the analysis of bibliometric variables and a qualitative approach to the study of recurring keywords, which has allowed us to demonstrate strands of literature that are not purely positive. There are currently some limitations that will affect future research potential, especially in ethics, data governance and the competencies of the health workforce.

## Data Availability

All the data are retrieved from public scientific platforms.
